# Changes in inhibitory control, craving and affect after yoga vs. aerobic exercise among smokers with nicotine dependence

**DOI:** 10.3389/fpsyt.2022.940415

**Published:** 2022-07-15

**Authors:** Hyungsook Kim, Jingu Kim, Minjung Woo, Teri Kim

**Affiliations:** ^1^Department of Cognitive Sciences, School of Intelligence, Hanyang University, Seoul, South Korea; ^2^Graduate School of Public Policy, Hanyang University, Seoul, South Korea; ^3^Hanyang Digital Healthcare Center, Hanyang University, Seoul, South Korea; ^4^Department of Physical Education, Kyungpook National University, Daegu, South Korea; ^5^School of Exercise and Sports Science, University of Ulsan, Ulsan, South Korea

**Keywords:** yoga, craving, affect, inhibition, smoking, Go/Nogo, ERP

## Abstract

**Objectives:**

This study investigated the acute effects of yoga and aerobic exercise on response inhibition and the underlying neural mechanisms in individuals with nicotine dependence, along with changes in craving and affect.

**Materials and methods:**

Study participants included 30 yoga-naïve adult smokers with moderate-to-high nicotine dependence. Based on a within-subjects design, all participants participated in three experimental sessions: baseline, 30-min yoga, and 30-min aerobic exercise; one session was conducted per day. The pre- and post-exercise Questionnaire of Smoking Urges and the Visual Analogue Scale were used to measure cigarette craving, and the Positive and Negative Affect Schedule was used to assess affective change. For cognitive measurement of inhibition, participants performed a Go/Nogo task consisting of Smoking-Go, Smoking-Nogo, Neutral-Go, and Neutral-Nogo stimulus conditions. Neuroelectric data were collected and the event-related potential (ERP) N2 and P3 amplitudes and latencies were analyzed.

**Results:**

Both yoga and aerobic exercise significantly reduced negative affect, whereas a reduction in craving was only observed after yoga. ERP results indicated that the P3 amplitudes after yoga were lower than those after aerobic exercise, suggesting increased neural efficiency after yoga, with reduced neural activity while maintaining the same level of cognitive performance as aerobic exercise.

**Conclusion:**

As yoga and aerobic exercise were equally effective in attenuating negative affect, smokers may expect greater benefits from yoga in craving reduction and inhibitory control with less physical and cognitive effort. We also believe that video-based yoga practice may provide additional benefits to these effects, reaching a large number of smokers in a non-face-to-face manner.

## Introduction

The prevalence of tobacco smoking is one of the most serious public health threats globally, claiming the lives of more than eight million people worldwide annually (1). Although several tobacco users are willing to quit after becoming aware of the specific health consequences of tobacco, smoking cessation has proven to be challenging, and only 4% of quit attempts are successful without professional support (1). Individuals addicted to nicotine often face various obstacles, such as withdrawal symptoms, nicotine craving, negative feelings, stress, and post-cessation weight gain (2, 3). Smoking relapse has been attributed to a higher level of craving and withdrawal symptoms upon initiating abstinence (4). Therefore, to increase the chances of successful cessation, evidence-based professional support is critical.

Exercise, a widely recommended intervention for smoking cessation, is easily accessible and cost-effective for the general population. Numerous studies have investigated the effects of various types of exercise on smoking cessation by examining how exercise affects craving and nicotine withdrawal symptoms. A recent meta-analysis that synthesized the existing randomized controlled trial (RCT) studies that investigated the effectiveness of exercise for smoking cessation—according to exercise type, aerobic exercise, and resisted exercise—found no effect on smoking cessation; however, yoga had a small positive effect (5). Yoga has a positive impact in terms of reducing craving, stress, and negative affect, and increasing positive affect, which are important predictors of smoking relapse (6, 7). Thus, yoga can be an effective treatment among various types of exercise.

Another systematic review on the effectiveness of aerobic exercise on smoking cessation incorporated the existing data from a total of 2,815 samples, and suggested that aerobic exercise was more effective than usual care (e.g., cognitive behavioral therapy and patient education) on smoking cessation in the short term, but did not demonstrate any differences in the long term (8). On the contrary, Wang et al. (9) meta-analysis reported that aerobic exercise and mind-body exercise can effectively attenuate withdrawal symptoms and reduce anxiety in individuals with nicotine addiction, without differences depending on exercise type (aerobic exercise vs. mind-body exercise) or levels of intensity (low, moderate, and high) (9). These conflicting findings illustrate the need for more robust controlled studies to ensure the quality of evidence of the effects of exercise on smoking cessation, which, in turn, would provide a scientific basis for the development and provision of optimized exercise therapy for smoking cessation.

Event-related potentials (ERPs), are increasingly used in both laboratory and clinical studies to investigate neural correlates of cognitive processes. ERPs are scalp-recorded voltage changes in the brain, derived from electroencephalographic (EEG) measurement, that are time-locked to the onset of specific stimuli (10). As a non-invasive measure with a high temporal resolution, ERPs are considered more objective than self-report measures and more sensitive than behavioral measures in that they provide information on the neural level (11). Therefore, ERPs are effective measures to capture internal changes or differences that might be too subtle to be externally manifested. The main components of ERP in the Go/Nogo inhibition paradigm are N2 and P3. N2 is a frontally distributed negative waveform arising within 200–300 ms time intervals after stimulus onset, while P3 is frontocentrally distributed positive waveform emerging 300–500 ms after stimulus onset (12, 13). Specifically, the inhibition-related signals (e.g., in response to Nogo trials) are thought to be generated by a distributed network involving the prefrontal areas, the anterior cingulate cortex, the motor areas and the parietal regions (14).

Nicotine dependence is closely linked to cognitive deficits and particularly relevant to response inhibition, a primary executive function that withholds undesired behavior or disregards task-irrelevant information, which may lead to smoking relapse when not functioning normally (15). Like other types of substance addiction, individuals with nicotine dependence perform worse on cognitive tests of inhibition, showing slower response times and/or lower accuracy than non-smoking controls during Go/Nogo tasks (16, 17). In addition to behavioral decline, ERP studies have also reported neurophysiological deficits in individuals with nicotine dependence, evidenced by reduced P3 with longer latencies (18), or reduced N2 amplitudes (19), which reflect cognitive impairments relevant to conflict monitoring and the suppression of automatic responses (12).

Although the effects of exercise on craving reduction are widely accepted, the effects of exercise on inhibitory control abilities associated with nicotine dependence are not clearly understood. An ERP study that investigated the effects of acute aerobic exercise on craving and inhibitory deficits in methamphetamine (MA) users found higher performance accuracy and larger N2 amplitudes during Go/Nogo tasks after an acute aerobic exercise session, accompanied by attenuated craving (13). Another ERP study that employed a brief mindfulness-mediation intervention reported that the mindfulness group demonstrated lower P3 amplitudes without behavioral differences when compared with controls, indicating less effortful response inhibition after mindfulness-based practice (12). Despite insufficient data, these previous studies suggest that aerobic exercise and a mindfulness-based intervention might have differential effects on neurophysiological measures of inhibitory control.

To our knowledge, this is the first study to directly compare the effects of yoga exercise and aerobic exercise on the neurophysiological indices of response inhibition in individuals with nicotine dependence. Based on previous findings that computer- or video-based smoking cessation programs could be an effective delivery strategy for participant motivation and adherence (20), this study aimed to explore the effectiveness of video-based yoga practice delivered non-face-to-face as compared to traditional aerobic exercise performed on a cycle ergometer. To provide a comprehensive understanding of the two exercise modes, we also examined changes in craving and positive and negative affect.

## Materials and methods

### Participants

Participants included 30 adult daily smokers (10 women, Mean Age = 25.97, Standard Deviation = 3.33) recruited *via* Internet advertisements posted on the website of K University, South Korea. Participants were screened by pre-determined eligibility criteria, which included (a) self-reported scores on the Fagerström test for nicotine dependence (FTND) of 4 or greater (to confirm moderate-to-high dependence) (21); (b) naïve to yoga or mindfulness-based exercises; (c) no contraindications for exercise assessed by the Physical Activity Readiness Questionnaire (PAR-Q) (22); (d) not currently participating in any smoking cessation program; (e) not currently using supplements—such as nicotine patches and gums or prescription drugs—for smoking cessation; (f) normal (or corrected-to-normal) vision and hearing; (g) no history of psychosis; and (h) no contraindications for electrophysiological measurements. The sample size required for this research design was estimated using the G*power calculator (version 3.1.9.4; Düsseldorf University, Düsseldorf, Germany) (23). The sample size was estimated based on a significance level of 0.05 and the statistical power of 0.95. We applied the effect size found in a previous study which employed a within-subjects, counterbalanced design to investigate acute exercise effects on craving and inhibitory control in methamphetamine-dependent individuals (ηp^2^ = 0.35) (13). As a result of the calculation, the total sample size of 23 participants was required to achieve a power of 0.95. Therefore, the number of participants in this study (*n* = 30) is expected to have sufficient statistical power. All participants provided written informed consent and were compensated with 50,000 Korean won (equivalent to 42 USD) in cash for their participation. The study protocol was approved by the ethics committee of the authors’ institute (KNU-2020-150).

### Instruments

All participants provided demographic information (i.e., age, sex, level of education, profession, marital status, etc.), as well as cigarettes smoked per day, amount of drinking, and hours of sleep. They also completed the FTND as a measure of nicotine dependence and the PAR-Q to confirm whether they had any physical or medical condition that would impede exercise engagement. To evaluate subjective craving to smoke, participants completed the Questionnaire of Smoking Urges (QSU-brief) (24). They were also instructed to indicate their current craving status on a 10 cm Visual Analogue Scale (VAS), which ranged from “no craving at all” to “extreme craving.” To measure affective changes according to the type of exercise, we used the Positive and Negative Affect Schedule-Expanded Form (PANAS-X), comprising 10 positive and 10 negative items (25). Using the Korean versions, we collected responses to the QSU-brief, VAS, and PANAS-X at four time points of the experiment: pre-aerobic exercise, post-aerobic exercise, pre-yoga, and post-yoga. All questionnaires were completed via Google Forms and participants registered their responses using a tablet PC. The responses were automatically stored on Google Drive upon submission. Regarding the reliability of the scales used in the present study, the Cronbach’s alpha values were found to be 0.76 for the FTND, 0.98 for the QSU-brief, 0.76 for the RPE, and 0.66 and 0.80 for the PANAS-X positive and negative affect, respectively. We also measured the Rating of Perceived Exertion (RPE) (26) at four time points (before exercise and 10, 20, and 30-min after exercise) and recorded the participants’ heart rates (HR) throughout the exercise sessions, including 5 min at rest before and after the exercise using the Polar H10 HR sensor.

### Cognitive task

To serve the aim of the present study, a smoking-related Go/Nogo task was developed by modifying the Go/Nogo paradigms used in previous studies (12, 13, 19). Participants were required to perform 600 trials of the Go/Nogo task segmented into three blocks, which presented either smoking-related or non-smoking-related (neutral) images. Each block consisted of 200 trials, including four types of stimuli (75 Smoking-Go, 25 Smoking-Nogo, 75 Neutral-Go, and 25 Neutral-Nogo). This means that the cognitive task comprised neutral stimuli and smoking-related stimuli in equal proportions and that Nogo trials accounted for 25% of the task. Participants were instructed to respond only to Go stimuli (i.e., blue-framed images) by pressing a button with the right index finger as quickly and accurately as possible, while withholding a response to the Nogo stimuli (i.e., yellow-framed images). The match between the frame color and the required response was counterbalanced across participants. The order of the stimulus content (smoking vs. neutral) was completely randomized, with the order of Go and Nogo trials quasi-randomized to ensure that, at most, four Go and two Nogo trials were consecutively presented. Prior to the actual task, participants were given a small set of practice trials involving 15 additional non-smoking images that were not included in the actual task. Each trial began with a fixation cross displayed for 400–500 ms, followed by a display of the stimulus on a gray background for 300 ms. Then, a blank screen was displayed for a random interval between 600 and 900 ms. Participants were given the opportunity to take a short break between the blocks. The total task duration, depending on the length of the breaks, was approximately 20 min.

### Exercise interventions

For the aerobic exercise session, participants were instructed to exercise on a stationary cycle ergometer (Monark 828E) at self-selected pace, while self-monitoring to stay within 60–70% of their estimated maximum HR (i.e., 206.9–0.67 × age). The aerobic exercise session comprised a 5-min warm-up, 20-min main exercise, and 5-min cool-down. The yoga session was developed for beginners and pre-recorded by a certified Hatha yoga instructor, comprising a 5-min opening meditation, 20 min devoted for yoga postures (asanas), and a 5-min closing meditation to include the contraction and relaxation of various muscle groups combined with regulated breathing. The asanas were performed in the following order: seated (Sukasana-Vajrasana-Baddha Konasana), prone (Bharmanasana-Bitilasana-Marjariasana-Balasana-Bhujangasana-Adhomukha Svanasana), standing (Uttanasana-Utthita Trikonasana), and supine (Supta Matsyendrasana) postures, with natural connecting and stretching movements in between. While listening to the instructions of the yoga video in a quiet room, participants were encouraged to focus on physical sensations and attend to their breathing. The order of the exercise sessions was counterbalanced across participants to minimize any effects of order or practice.

### Procedure

The participants visited the laboratory on three non-consecutive days (mean = 47 ± 37.80 h between sessions) and individually engaged in the experimental condition: baseline, aerobic exercise, and yoga. Participants were instructed to abstain from smoking, consuming alcohol or caffeine, or engaging in any form of exercise for at least 12 h before the experiment. On the first visit for baseline testing, the participants were provided information regarding the study, including the procedure, equipment, and cognitive tests. After having the opportunity to ask questions, they provided written informed consent. Participants were then instructed to answer demographic questions and complete the FTND, PAR-Q, and International Physical Activity Questionnaire (IPAQ) (27). After completing the set of self-report measures, participants were fitted with electrode caps and seated in a light and sound-attenuated room; thereafter, they performed the baseline Go/Nogo task. They returned for subsequent testing after the baseline cognitive measurement. On two separate visits for the yoga and aerobic exercise sessions, participants were first instructed to complete the pre-exercise QSU-brief, VAS, and PANAS-X. Thereafter, they wore a chest strap HR monitor and were led to the exercise room to perform either yoga or aerobic exercise for 30 min. For the yoga session: A laptop, connected to a 65 cm × 112 cm standing monitor (LH50BEA, Samsung), was used, and participants played the video themselves once they were ready to begin. To measure perceived exertion, pre-recorded messages were displayed at four time points (beginning, 10-min past, 20-min past, and completion) to guide the participants to pause and respond to RPE using the paper and pencil in front of the screen. For the aerobic exercise session, participants self-administered the cycle ergometer exercise for 30 min, including a 5-min warm-up and 5-min cool-down. For the remaining 20 min, participants were asked to aim to maintain their HR between 60 and 70% of the individual maximum HR. A research assistant collected responses to the RPE at four time points. Immediately following the completion of the exercise session, participants were escorted to the preparation room, where they completed the post-exercise QSU-brief, VAS, and PANAS-X and were prepared for electroencephalogram (EEG) measurement. Thereafter, participants were led to the cognitive testing room to perform the Go/Nogo task. There were 2- to 3-day intervals between the three visits.

### Electroencephalographic recording and processing

Electroencephalographic data were obtained using the Biopac MP150 system (Biopac Systems Inc., Santa Barbara, CA, United States) run by AcqKnowledge 5.0 software (Biopac Systems Inc.). An elastic EEG cap (CAP100C, Biopac Systems Inc.) was used with the following electrodes arranged in accordance with the international 10–20 system: Fp1 (left prefrontal), Fp2 (right prefrontal), F3 (left frontal), Fz (mid frontal), F4 (right frontal), C3 (left central), Cz (mid central), C4 (right central), P3 (left parietal), Pz (mid parietal), and P4 (right parietal) (28). Continuous EEG and electrooculogram (EOG) signals were recorded at a sampling rate of 1000 Hz with a bandpass of 0.01–100 Hz, online-referenced against linked mastoids, with Fpz serving as the ground electrode. For all channels, impedance levels were maintained below 5 kΩ using a Grass Impedance Meter (EZM5, Astro-Med Inc., West Warwick, RI, United States). Prior to further data analyses, EEG signals with amplitudes exceeding ± 100 μV or contaminated by artifacts or eye blinks (EOG) were inspected and excluded. We performed EEG data analysis using a MATLAB-based EEG analysis program. Baseline corrections were conducted using the mean 200 ms pre-stimulus period, and the data were bandpass-filtered (0.1–30 Hz; 24 dB/octave). To extract a stimulus-locked epoch, data were segmented into epochs of 1 s (200 ms prior to and 800 ms following stimulus onset). Segments of incorrect responses (i.e., miss for Go trials or false alarms for Nogo trials) were excluded from the analyses. Three representative electrodes (i.e., Fz, Cz, and Pz) were chosen for analysis, identifying responses from frontal, central, and parietal regions, respectively, based on previous Go/Nogo studies that have predominantly examined and observed inhibition-related N2 and P3 effects at Fz, Cz, and Pz (11).

### Statistical analysis

This study was based on a counterbalanced within-subject study design. To examine participants’ craving to smoke, we analyzed the QSU-brief and VAS data separately using 2 (exercise: aerobic exercise, yoga) × 2 (time point: pre, post) repeated-measures analyses of variance (RM-ANOVAs). For the PANAS-X responses, the positive mood and negative mood scores were separately analyzed using 2 (exercise: aerobic exercise, yoga) × 2 (time point: pre, post) RM-ANOVAs. To determine whether different modes of exercise resulted in any differences in the accuracy and response time (RT) for the Go/Nogo task performance, separate 3 (exercise: baseline, aerobic exercise, yoga) × 4 (stimulus: Smoking-Go, Smoking-Nogo, Neutral-Go, Neutral-Nogo) RM-ANOVAs were performed. Regarding the neuroelectric data, separate 3 (exercise: baseline, aerobic exercise, yoga) × 4 (stimulus: Smoking-Go, Smoking-Nogo, Neutral-Go, Neutral-Nogo) × 3 (electrode site: Fz, Cz, Pz) RM-ANOVAs were applied to analyze the ERP data of N2 amplitude, N2 latency, P3 amplitude, and P3 latency. For the analysis of exercise intensity, 2 (exercise: aerobic exercise, yoga) × 3 (time point: pre, mid, post) RM-ANOVAs were performed for the HR mean, while the RPE data were analyzed using 2 (exercise: aerobic exercise, yoga) × 4 (time point: pre, 10, 20, 30-min) RM-ANOVA. All statistical analyses were performed using SPSS 22.0, and an alpha value of 0.05 was set as the significance level, where p-values were Greenhouse–Geisser adjusted when appropriate. *Post hoc* analyses were conducted using pairwise comparisons with Bonferroni adjustments.

## Results

### Manipulation check: Heart rates and rating of perceived exertion

Analysis of the mean HR measured before, during, and after each exercise session revealed significant main effects of exercise mode [*F*_(1,29)_ = 97.068, *p* = 0.000, partial ηp^2^ = 0.770], time point [*F*_(2,58)_ = 111.081, *p* = 0.000, partial ηp^2^ = 0.799], and an interaction effect between exercise mode and time point [*F*_(2,58)_ = 76.415, *p* = 0.000, ηp^2^ = 0.725]. *Post hoc* analyses revealed that aerobic exercise exhibited a higher mean HR than yoga during and after exercise. In terms of self-reported exercise intensity, RPE responses yielded a significant main effect of exercise mode [*F*_(1,29)_ = 14.636, *p* = 0.001, ηp^2^ = 0.335], time point [*F*_(3,87)_ = 94.725, *p* = 0.000, ηp^2^ = 0.766], and an interaction effect between exercise mode and time point [*F*_(3,87)_ = 10.016, *p* = 0.000, ηp^2^ = 0.257]. *Post hoc* analysis revealed that the RPE of aerobic exercise measured after 10 min and post-exercise was higher than that of yoga.

### Assessment of craving and affect

In the QSU-brief analysis, no significant differences emerged as a function of exercise mode or measurement time. However, the VAS results indicated a significant main effect of exercise mode [*F*_(1,29)_ = 6.146, *p* = 0.019, ηp^2^ = 0.175] and an interaction effect between exercise mode and time point [*F*_(1,29)_ = 7.701, *p* = 0.043, ηp^2^ = 0.133]. Specifically, the VAS score was lower after yoga than after aerobic exercise, without a baseline difference. Regarding the measures of affective change, the PANAS-positive mood score revealed no significant difference depending on exercise mode and time point. However, the PANAS-negative mood score yielded a significant main effect of time point [*F*_(1,29)_ = 13.524, *p* = 0.001, ηp^2^ = 0.318], indicating that both yoga and aerobic exercise reduced post-exercise negative mood compared to pre-exercise. The main effect of exercise type and interaction effect between exercise mode and time point were not statistically significant (Table 1).

**TABLE 1:** Differences in craving, affect, cognitive performance, and exercise intensity.

		Baseline		Aerobic exercise		Yoga	
		Mean	95% CI	Mean	95% CI	Mean	95% CI
Question of smoking urges-brief	Pre-exercise			36.87	34.23–39.51	35.67	32.75–38.59
	Post-exercise			36.97	31.42–38.52	32.33	28.89–35.77
Visual analogue scale	Pre-exercise			7.42	6.67–8.18	7.26	6.57–7.95
	Post-exercise			7.26	6.33–8.20	6.09	5.18–7.00
PANAS-positive	Pre-exercise			22.83	20.81–24.86	22.57	19.88–25.25
	Post-exercise			23.37	20.63–26.10	23.13	20.15–26.12
PANAS-negative	Pre-exercise			15.70	13.68–17.72	15.27	13.57–16.97
	Post-exercise			14.03	11.96–16.11	12.86	11.42–14.32
Response time (ms)	Smoking-Go	411.51	378.07–444.95	396.34	362.90–429.78	402.45	369.01–435.89
	Neutral-Go	409.79	376.35–443.23	400.22	366.78–433.69	395.27	361.83–428.71
Accuracy (%)	Smoking-Go	91.56	86.49–96.62	90.64	85.57–95.71	89.67	84.61–94.74
	Smoking-Nogo	86.22	81.15–91.29	85.56	80.49–90.62	85.82	80.75–90.89
	Neutral-Go	82.87	77.81–87.94	82.07	77.01–87.14	86.41	81.75–90.89
	Neutral-Nogo	92.36	87.29–97.42	89.33	84.27–94.40	89.91	84.84–94.98
Heart rate (bpm)	Pre-exercise			79.10	75.31–82.89	78.70	74.78–82.62
	Mid-exercise			116.67	112.15–121.18	81.27	77.03–85.51
	Post-exercise			88.23	84.23–92.24	73.93	70.55–77.32
Ratings of perceived exertion	Pre-exercise			7.033	6.27–7.80	7.10	6.17–8.03
	10-min past			12.60	11.41–13.79	9.30	8.38–10.22
	20-min past			14.53	13.48–15.58	13.57	12.84–14.29
	Post-exercise			14.17	13.01–15.32	11.67	10.68–12.65

### Behavioral measures

**FIGURE 1:**
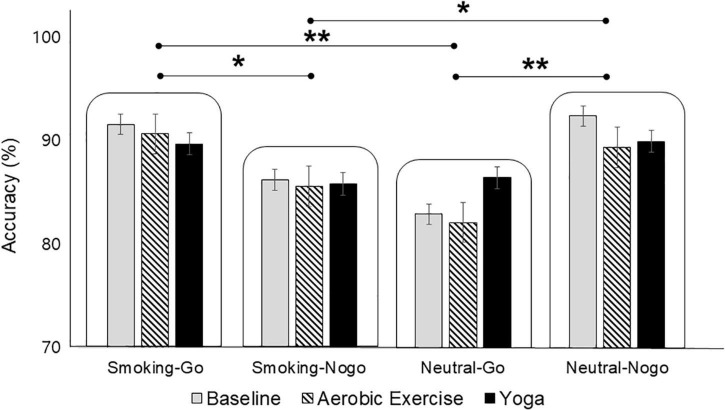
Differences in Go/Nogo task performance accuracy as a function of stimulus condition. *p < 0.05; **p < 0.01.

Separate 3 (exercise: baseline, aerobic exercise, yoga) × 4 (stimulus: Smoking-Go, Smoking-Nogo, Neutral-go, Neutral-Nogo) RM-ANOVAs were performed to examine any differences in the accuracy and response time of the Go/Nogo task performance as a function of exercise and/or stimulus conditions. In the accuracy analysis, significant main effects of the stimulus [*F*_(3,348)_ = 5.306, *p* = 0.001, ηp^2^ = 0.044] emerged. *Post hoc* tests revealed higher accuracy on the Smoking-Go and Neutral-Nogo tasks than on the Smoking-Nogo and Neutral-Go tasks (*p* < 0.05, Figure 1). Neither a main effect of exercise mode nor interaction effects between exercise mode and stimulus condition emerged (*p* > 0.05). In the analysis of response time as a function of exercise and stimulus, neither the main effects nor interactions were statistically significant (*p* > 0.05).

### Neuroelectric measures

#### N2 mean amplitude and latency

**FIGURE 2:**
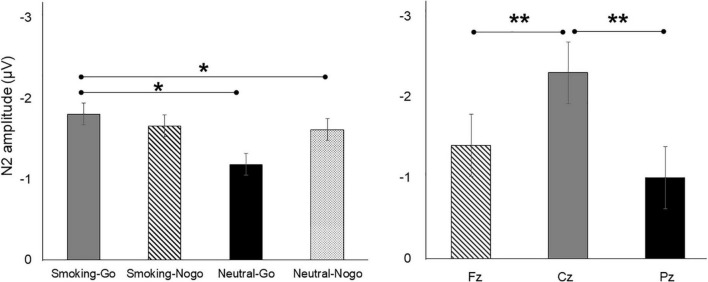
Differences in N2 amplitudes as a function of stimulus condition and region. *p < 0.05; **p < 0.01.

Three-way RM-ANOVA evaluating the N2 amplitude revealed significant main effects of the stimulus [*F*_(3,1044)_ = 3.043, *p* = 0.028, ηp^2^ = 0.009] and electrode sites [*F*_(2,1044)_ = 14.521, *p* = 0.000, ηp^2^ = 0.027]. The *post hoc* comparison indicated that the Smoking-Go stimulus was associated with greater N2 amplitudes than the Neutral-Go (*p* = 0.029) and Neutral-Nogo stimuli (*p* = 0.012), while greater N2 amplitudes were elicited at Cz than at Fz (*p* = 0.000) and Pz (*p* = 0.000, Figure 2). However, the main effect of exercise mode was not significant, with no interaction effects observed among exercise modes, stimulus conditions, and electrode sites (*p* > 0.05). Analysis of N2 latency revealed no significant effects (*p* > 0.05).

#### P3 mean amplitude and latency

**FIGURE 3:**
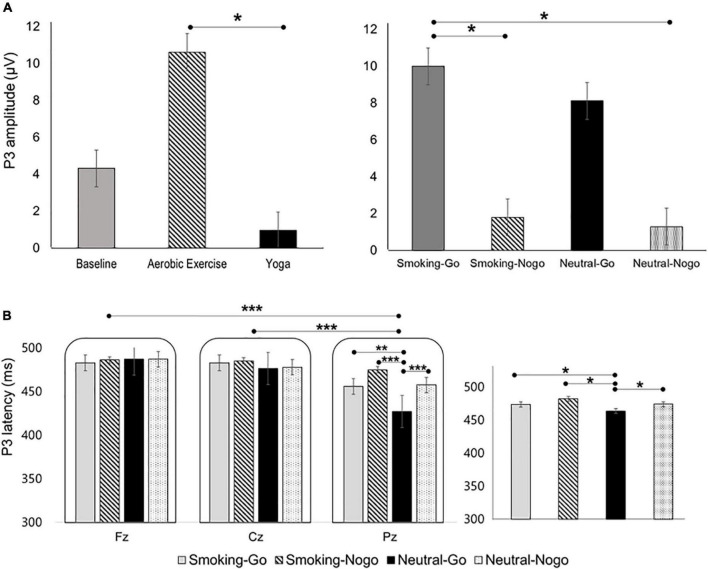
Differences in **(A)** P3 amplitudes and **(B)** latencies as a function of exercise mode, stimulus condition, and region. *p < 0.05; **p < 0.01; ***p < 0.001.

Regarding P3 amplitude, three-way RM-ANOVA revealed significant main effects of exercise [*F*_(2,1044)_ = 4.476, *p* = 0.012, ηp^2^ = 0.009] and stimulus [*F*_(3,1044)_ = 2.726, *p* = 0.043, ηp^2^ = 0.008]. *Post hoc* comparisons indicated that the P3 amplitude after aerobic exercise was higher than that after yoga (*p* = 0.010), while the Smoking-Go condition was associated with a higher P3 amplitude compared to Smoking-Nogo (*p* = 0.030) and Neutral-Nogo (*p* = 0.021; Figure 3A). The main effect of electrode sites and interaction effects on P3 amplitude were not statistically significant (*p* > 0.05).

**FIGURE 4:**
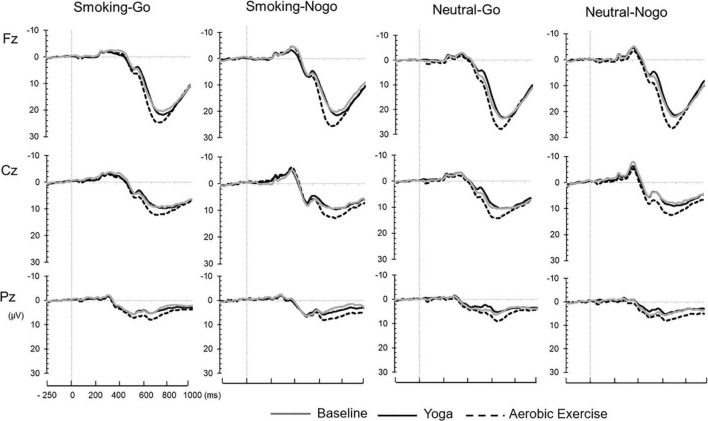
Grand-averaged ERP waveforms.

The three-way RM-ANOVA evaluating P3 latency revealed significant main effects of stimulus [*F*_(3,1044)_ = 5.816, *p* = 0.001, ηp^2^ = 0.016], electrode site [*F*_(2,1044)_ = 40.499, *p* = 0.000, ηp^2^ = 0.072], and an interaction between stimulus and electrode sites [*F*_(6,1044)_ = 4.100, *p* = 0.000, ηp^2^ = 0.023]. *Post hoc* tests indicated that the Neutral-Go condition was associated with shorter P3 latency than the Smoking-Go (*p* = 0.001), Smoking-Nogo (*p* = 0.000), and Neutral-Nogo (*p* = 0.000) conditions, while the P3 latency was shorter at Pz than at Fz (*p* = 0.000) and Cz (*p* = 0.000, Figure 3B). Regarding the interaction effects, a shorter P3 latency of the Neutral-Go condition compared to Smoking-Go (*p* = 0.001), Smoking-Nogo (*p* = 0.000), and Neutral-Nogo (*p* = 0.000) was observed at Pz. The main effect of exercise and other interactions on P3 latency were not significant (*p* > 0.05). The grand-averaged waveforms collapsed across stimulus conditions for baseline, yoga, and aerobic exercise at the Fz, Cz, and Pz electrode sites are presented in Figure 4.

## Discussion

### Craving and affect

Regarding the measures of cigarette craving, the descriptive data of the QSU-brief revealed an 12.55% reduction after yoga and 3.25% after aerobic exercise, but the differences according to exercise modes and measurement time were not statistically significant. These findings do not support that of a previous study by Elibero et al. (6), which reported that 30-min yoga session delivered by instructional DVD and aerobic exercise performed on a treadmill both significantly reduced the QSU-brief score, where exercise intensity was lower for yoga than for aerobic exercise. However, the VAS results in our study indicated a significant reduction in craving only after yoga, whereas aerobic exercise exhibited no pre-post craving reduction. Despite the inconsistency in previous findings, the craving measures in the present study suggest the possibility that light-intensity yoga, including meditation and focused breathing, might be more effective than moderate-intensity aerobic exercise in reducing cigarette craving. Due to the scarcity of evidence that directly compares the acute effects of yoga to aerobic exercise on craving, sufficient follow-up studies are required to obtain more generalizable results.

As measured by PANAS-X, negative affect was significantly reduced after both aerobic exercise and yoga, without any significant changes in positive affect. These results are in line with previous studies reporting the positive effects of exercise on at least one measure of affect (29). In a previous study that examined the effects of 10 min of moderate-intensity ergometer exercise on smoking withdrawal symptoms, positive and negative affect outcomes were not significantly correlated, while negative affect was more closely correlated with other factors, such as irritability, depression, tension, restlessness, difficulty concentrating, and stress (30). Furthermore, the affective benefits of exercise are independent of exercise intensity, whereas cognitive control function might be modulated by exercise intensity (31). These previous findings support the present study’s affective outcomes: Both light-intensity yoga and moderate-intensity aerobic exercise attenuated negative affect without significantly changing the positive affect measures. Regarding energy expenditure, yoga may help reduce negative affect in smokers with less physical demand while having a greater effect on craving reduction.

### Cognitive performance

The behavioral outcomes of the cognitive task demonstrated that participants were more accurate on Smoking-Go than on Smoking-Nogo tasks, while they were more accurate on Neutral-Nogo than on Neutral-Go tasks. Consistent with previous studies on inhibitory control among smokers, in which more execution errors (i.e., executing a response to a Nogo target) were observed in smoking-related backgrounds than in neutral backgrounds (32), the participants in the present study also exhibited deficits in suppressing responses to smoking-related cues as compared to neutral cues (33). Reportedly, substance abusers have impaired cognitive control function and demonstrate a greater level of attention toward substance-related cues (19, 34). Therefore, considering that the present study’s participants were regular smokers with moderate- to high-nicotine dependence, the lower accuracy observed in Smoking-Nogo relative to Neutral-Nogo tasks concurs with the reduced inhibitory control observed in individuals with addiction during substance-related cue exposure.

The differences in performance accuracy also illustrate that when presented with familiar or favorable (i.e., smoking-related) cues, participants found it easier to execute (i.e., Go)—than to withhold (i.e., Nogo)—a response. However, when presented with neutral stimuli, which might have been less familiar or less favorable, response execution might have been more difficult than inhibition. This can be understood in terms of the association with the approach and withdrawal motivation: That is, decision-making can be primarily influenced by the valence of the stimulus, generating action or approach in positive contexts, while promoting inhibition or withdrawal in negative contexts (35). Given participants’ high nicotine dependence, it is possible that smoking cues, accepted as emotionally more positive than neutral cues, better facilitated approach motivation for Smoking-Go trials as compared to Smoking-Nogo trials; conversely, the neutral cues, attracting relatively less attention, activated withdrawal motivation, thereby leading to better performance on Neutral-Nogo trials than Neutral-Go trials.

Contrary to our expectations, no significant differences emerged in the behavioral measures as a function of the exercise mode. The absence of behavioral differences between aerobic exercise and yoga might be associated with the elapsed time between exercise completion and cognitive measurement, which caused a 10-min delay (approximately). A bout of exercise for 30 min might have been insufficient to sustain the exercise effects through the experiment and precipitate behavioral enhancement. Therefore, since the immediate effect of exercise may not have been externally manifested as behavioral outcomes of cognitive task performance, we employed ERP measures, which are internal and sensitive indicators of cognitive processes, to identify the effects caused by different exercise modes.

### Event-related potential measures

The ERP N2 component in a visual Go/Nogo paradigm represents cognitive control processes related to conflict monitoring and response inhibition (36). As a neurophysiological marker associated with nicotine dependence, impaired cognitive control function has been evidenced by abnormal N2, with smaller Nogo-N2 amplitudes observed in smokers than in non-smokers (34, 37). However, caution should be employed when generalizing this interpretation because the non-smoker control group was not included in the design of the present study. Whether N2 is an index of inhibition or conflict monitoring processes is contentious (38); however, both interpretations suggest that N2 reflects a cognitive control process. Therefore, a reduced N2 implies a less active conflict-detection process. In the present study, greater N2 amplitudes were associated with Smoking-Go tasks than with Neutral tasks. This suggests that the individuals with nicotine dependence in the present study demonstrated more active conflict detection for the Smoking-Go stimuli, which might have led to higher accuracy in Smoking-Go trials. A relatively smaller N2 was associated with the Neutral-Go and Neutral-Nogo conditions. This may suggest that the cognitive efforts related to conflict monitoring or inhibition—compared to the smoking-related stimulus—decreased in the neutral contexts, presumably due to reduced motivation or interest. This interpretation is supported by a recent study by Chen et al. (39), in which individuals with Internet addiction demonstrated larger N2 and P3 toward Internet-related cues than toward irrelevant cues, reflecting a positive motivational implicit response. Additionally, the N2 amplitude was maximal in the central midline region (i.e., Cz) than in the frontal and parietal midline regions (Fz and Pz). Considering that the N2 in the visual Go/Nogo task is usually the largest over frontocentral scalp sites approximately 250–350 ms after stimulus onset (40), the N2 observed in our study, peaking at Cz, is not an unexpected result.

Notably, P3 amplitudes were greater after aerobic exercise than after yoga. The P3 component reflects cognitive processes associated with inhibition, attention allocation, and the working memory (41). The enhanced P3 after aerobic exercise relative to yoga can be explained in terms of the exercise intensity. The HR and RPE measured in the present study showed that aerobic exercise intervention corresponded to moderate intensity (HR mean = 116.67, SD = 12.08), whereas the yoga intervention was performed at light intensity (HR mean = 81.27, SD = 11.35). The RPE responses also indicated significant differences after 10 min of exercise and at the end of exercise: The participants perceived aerobic exercise as somewhat hard and yoga as fairly light exercise (42). Prior research illustrated an inverted-U-shape relationship between exercise intensity and P3 amplitude (43), given the correlations between physical arousal and performance and the fact that P3 is affected by biological processes, such as the exerciser’s arousal state (42, 44). Specifically, the P3 amplitude decreased and increased after high- and medium-intensity pedaling exercise, respectively; in contrast, there was no change after low-intensity pedaling exercise (43). Therefore, greater P3 after aerobic exercise than after yoga might be linked to the influence of exercise intensity. Similarly, Wang et al. (9) reported an inverted-U-shaped relationship between exercise intensity and inhibitory control both in behavioral and neuroelectric indices, wherein participants with methamphetamine dependence exhibited higher accuracy, faster RT, and greater N2 amplitudes after a short bout of moderate-intensity exercise compared to low-intensity exercise and inactive control (41).

Contrary to previous studies that tested varying exercise intensities using the same exercise mode, the aerobic exercise and yoga interventions used in our study were different in nature. Furthermore, we found no significant differences between aerobic exercise and yoga at the behavioral level in terms of accuracy and RT. Therefore, the greater P3 cannot be interpreted as evidence that aerobic exercise has greater cognitive benefits than yoga. The cognitive task performance was, though not statistically significant, generally higher after yoga than after aerobic exercise; thus, the possibility exists that the smaller P3 after yoga indicates higher neural efficiency, rather than attentional disadvantage associated with the nature of the particular type of exercise. This interpretation is supported by growing evidence of the effects of mindfulness practice on the P3 component. Andreu et al. (12) reported smaller P3 amplitudes in cigarette smokers during the Nogo task after a bout of mindfulness practice compared with controls—without behavioral level differences (26). Here, a smaller P3 indicated less effortful inhibitory control to reach a similar level of performance as the control group (45, 46).

Yoga offers a way to practice mindful awareness (47). During yoga practice in the present study, participants were guided into a pose, encouraged to deepen their breath, and to witness their experience non-judgementally (i.e., to be mindful). Taking a meta-cognitive view of the present experience through mindfulness practice is thought to strengthen self-regulation (48). Based on the evidence from mindfulness studies, Gard et al. (48) hypothesized that yoga practice over time changes the neural processes that sub-serve self-regulation to be more automatized and efficient. According to Thayer and Lane (49), continued mindfulness practice integrates inhibitory control mechanisms with autonomic, attentional, and affective systems, creating a functional and structural network for self-regulation. Consequently, appraisal processes become more implicit and efficient, which facilitates inhibition between old maladaptive responses and newer, more goal-directed responses (49). However, since the evidence for the effects of yoga on the inhibitory process is scarce, the scientific basis supporting such interpretations largely depends on mindfulness studies. In that respect, this study is the first attempt to provide empirical evidence that yoga improves inhibitory control function, which has only been theoretically explained in previous studies. Furthermore, the present study found such an effect after a single session of yoga, while previous studies suggested it as a result of continued practice over time (48).

In the longer term, reductions in P3 amplitude and/or shorter P3 latency have been observed after 8 weeks (50) and 3 months (51) of mindfulness meditation training, as well as in experienced meditators (52), reflecting enhanced efficiency of attentional resource allocation. These findings support the interpretation of the lower P3 after yoga relative to aerobic exercise in our study, which indicates more efficient cognitive processing, implying that more cognitive endeavors are required to complete the inhibition task after aerobic exercise than after yoga (53). However, this explanation is still not straightforward because of the scarcity of previous research comparing the effects of yoga and aerobic exercise on cognitive function. More direct relationships between the P3 component and yoga should be investigated in future research.

Analyses of the P3 amplitude also revealed that compared with the Smoking-Nogo and Neutral-Nogo trials, the Smoking-Go trials were associated with greater P3. This result is consistent with a previous study that reported a larger P3 for targets than non-targets (54). Additionally, P3 is affected by task relevance and probability (55). Therefore, the larger P3 amplitudes during the Smoking-Go trials in our study may indicate that the participants were more attentive to cigarette-related stimuli requiring an overt response. The P3 measurements in this study exhibited a general pattern of larger amplitudes with shorter latencies for the Go trials and smaller amplitudes with prolonged latencies for the Nogo trials. This can be explained by our study participants’ characteristics. Reduced Nogo-P3 amplitudes and longer Nogo-P3 latencies have frequently been reported in cigarette smokers (56) as well as individuals with other types of substance dependence (57–59). Therefore, in our study, reduced P3 amplitudes with longer latencies during Nogo trials seem to reflect attentional deficiency in the later stage of the inhibitory process and processing speed as general characteristics associated with nicotine dependence.

In summary, this study investigated the effects of acute yoga versus aerobic exercise on craving, affect, and inhibitory control function in individuals with nicotine dependence. Self-reported measures indicated attenuated cigarette craving after yoga but not after aerobic exercise, and both exercises were effective in reducing negative affect. The behavioral results revealed no significant differences based on the mode of exercise—both in terms of accuracy and RT. However, the neuroelectric data indicated that the P3 amplitude was lower after yoga than after aerobic exercise, which may reflect a more efficient inhibitory control after yoga associated with the mindful and contemplative nature of the exercise.

### Limitations

This study had several limitations. First, since we applied a within-subject study design, the findings of our study lack comparisons with non-smokers. We recommend that future studies compare the effects of yoga and aerobic exercise on inhibitory control processes between smokers and non-smokers. The neurophysiological outcomes of this study might not reflect the immediate effect of exercise, as we failed to control the delay between exercise completion and cognitive measurement, which might have diminished the immediate effect of exercise. Furthermore, differences in exercise intensity (i.e., light-intensity yoga vs. moderate-intensity aerobic exercise) and delivery (i.e., video-guided yoga vs. self-administered aerobic exercise) might have affected the results. As the study participants were naïve to yoga, they might have been relatively more familiar with aerobic exercise than yoga, thus potentially affecting their exercise engagement. In the present study, the acute exercise sessions yielded significant differences only at the neurophysiological level and not at the behavioral level. Therefore, future studies should employ an extended duration of interventions to confirm whether the positive effects of exercise are behaviorally manifested and further investigate differences in delayed effects, adherence, and abstinence—according to varying exercise modes.

### Implications

To the best of our knowledge, this is the first study to examine the acute effects of yoga compared with aerobic exercise on inhibitory control processes using ERPs. A 30-min video-based yoga practice resulted in increased neural efficiency, exhibiting reduced neural activity while maintaining the same level of cognitive performance as a 30-min aerobic exercise. The enhanced neural efficiency after a short bout of yoga may be attributed to reduced craving. Given that yoga was as effective as aerobic exercise in attenuating negative affect, smokers may expect greater benefits from yoga in terms of craving reduction and inhibitory control—with less effortful physical and cognitive exertion. Contrary to previous findings where moderate and vigorous intensity exercises were more effective in craving reduction than light intensity exercise, the present study observed a significant craving reduction only after yoga, which was performed at light intensity (13). Future studies need to investigate the effects of yoga and aerobic exercise on craving, affect, and cognitive function with intensity control to confirm the findings of the present study. Considering the overall features of behavioral and neuroelectric outcomes, yoga appears to be a highly effective exercise option for smokers who need assistance with response inhibition and craving reduction. We also suggest that video-based yoga practice potentially benefits these effects by reaching a large number of smokers in a non-face-to-face manner.

## Data availability statement

The original contributions presented in this study are included in the article/supplementary material, further inquiries can be directed to the corresponding author.

## Ethics statement

The studies involving human participants were reviewed and approved by Institutional Review Board of Kyungpook National University. The patients/participants provided their written informed consent to participate in this study.

## Author contributions

JK and TK contributed to conception and design of the study. MW and TK collected the data and performed the (statistical) analysis. TK wrote the first draft of the manuscript. HK, JK, and MW wrote sections of the manuscript. HK contributed to funding acquisition. All authors contributed to manuscript revision, read, and approved the submitted version.

## Conflict of interest

The authors declare that the research was conducted in the absence of any commercial or financial relationships that could be construed as a potential conflict of interest.

## Publisher’s note

All claims expressed in this article are solely those of the authors and do not necessarily represent those of their affiliated organizations, or those of the publisher, the editors and the reviewers. Any product that may be evaluated in this article, or claim that may be made by its manufacturer, is not guaranteed or endorsed by the publisher.
